# Pain manifestations in nursing professionals: a scoping review

**DOI:** 10.17533/udea.iee.v43n1e13

**Published:** 2025-04-29

**Authors:** Sabrina de Melo Oliveira, Larissa de Lima Ferreira, Thaís Araújo da Silva, Iracema da Silva Frazão

**Affiliations:** 2 Nurse, Master student. Email: larissa.limaf@ufpe.br https://orcid.org/0000-0003-1024-4511 Universidade Federal de Pernambuco Brazil larissa.limaf@ufpe.br; 3 Nurse, Ph.D. student. Email: thais.araujosilva@ufpe.br https://orcid.org/0000-0001-8408-6857 Universidade Federal de Pernambuco Brazil thais.araujosilva@ufpe.br; 4 Nurse, Ph.D. Email: iracema.frazao@ufpe.br https://orcid.org/0000-0002-1218-9096 Universidade Federal de Pernambuco Brazil iracema.frazao@ufpe.br; 5 Federal University of Pernambuco (UFPE), Recife/PE, Brazil. https://orcid.org/0000-0002-4690-3753 Universidade Federal de Pernambuco Federal University of Pernambuco (UFPE) Recife/PE Brazil

**Keywords:** pain, nursing professionals, occupational diseases., dolor, enfermeras practicantes, enfermedades profesionales., dor, profissionais de enfermagem, doenças profissionais

## Abstract

**Objective.:**

To map the literature on the prevalence of pain in nursing professionals.

**Methods.:**

This is a scoping review that was conducted according to the Joanna Briggs Institute (JBI) methodology for scoping reviews, and according to the Preferred Reporting Items for Systematic Reviews and Meta-Analyses extension for Scoping Reviews (PRISMA-ScR). The protocol was developed and registered in the Open Science Framework (OSF) [https://osf.io/2zu73/]. The search was carried out in the following databases: PubMed/MEDLINE, Virtual Health Library (VHL), Web of Science, Scientific Electronic Library Online (SciELO), SciVerse Scopus, Embase, and the Catalog of Theses and Dissertations of the Coordination for the Improvement of Higher Education Personnel (CAPES).

**Results.:**

A total of 49 studies were included, all of which were cross-sectional studies, and the total sample of the included studies was 35,069 participants. Most of the included studies were concentrated in the Asian continent (71.4%). Among the selected studies, it was shown that the most affected area was the lumbar region (81.57%), followed by the neck (71.5%) and shoulder (31.57%) regions.

**Conclusion.:**

According to the studies evaluated, the prevalence of occupational pain in nursing professionals was of musculoskeletal origin. The high prevalence of pain found reinforces the importance of monitoring the health of nursing workers.

## Resumen

**Objetivo.:**

Mapear la literatura científica sobre la prevalencia del dolor en los profesionales de enfermería.

**Métodos.:**

Se trata de una revisión de alcance que se llevó a cabo de acuerdo con las metodologías del Instituto Joanna Briggs y con la extensión de la guía PRISMA. El protocolo se elaboró y registró en el Open Science Framework (OSF) [https://osf.io/2zu73/]. La búsqueda se realizó en las siguientes bases de datos: PubMed/MEDLINE, Biblioteca Virtual de Salud (BVS), Web of Science, Scientific Electronic Library Online (SciELO), SciVerse Scopus, Embase y el Catálogo de Tesis y Disertaciones de la Coordinación para el Perfeccionamiento del Personal de Educación Superior (CAPES).

**Resultados.:**

Se incluyeron 49 estudios, todos eran estudios transversales, incluyendo la muestra un total de 35069 participantes. La mayoría de los estudios incluidos se concentraban en el continente asiático (71.4%). Entre los estudios seleccionados, se encontró que la zona más afectada por el dolor era la región lumbar (81.57%), seguida de las regiones de cuello (71.5%) y hombros (31.57%).

**Conclusión.:**

Según los estudios evaluados, la prevalencia de dolor ocupacional en los profesionales de enfermería fue de origen musculoesquelético. La elevada prevalencia de dolor encontrada refuerza la importancia de acompañar la salud de los trabajadores de enfermería.

## Resumo

**Objetivo.:**

Mapear a literatura acerca da prevalência de dor em profissionais de enfermagem.

**Métodos.:**

Trata-se de uma revisão de escopo que foi conduzida de acordo com metodologia do Joanna Briggs Institute (JBI) para revisões de escopo, e de acordo com o *Preferred Reporting Items for Systematic Reviews and Meta-Analyses extension for Scoping Reviews* (PRISMA-ScR). O O protocolo foi elaborado e registrado no Open Science Framework (OSF) [https://osf.io/2zu73/]. A pesquisa foi realizada nas bases de dados: PubMed/MEDLINE, Biblioteca Virtual em Saúde (BVS), Web of Science, *Scientific Electronic Library Online* (SciELO), SciVerse Scopus, Embase e o Catálogo de Teses e Dissertações da Coordenação de Aperfeiçoamento de Pessoal de Nível Superior (CAPES).

**Resultados.:**

Foram incluídos 49 estudos, todos eram estudos transversais e a amostra total dos estudos incluídos foi de 35.069 participantes. Maior parte dos estudos incluídos se concentrou no continente asiático (71.4%). Dentre os estudos selecionados, demonstrou-se que a área mais afetada foi a região lombar (81.57%), seguidas pelas regiões do pescoço (71.5%) e ombro (31.57%).

**Conclusão.:**

De acordo com os estudos avaliados, a prevalência de dor ocupacional nos profissionais de enfermagem foi de origem musculoesquelética. A alta prevalência de dor encontradas reforça a importância do acompanhamento da saúde dos trabalhadores de enfermagem.

## Introduction

Pain is defined by the International Association for the Study of Pain (IASP) as “an unpleasant sensory and emotional experience associated with, or similar to, that associated with actual or potential tissue damage.” ^1^ It is one of the most common symptoms leading to medical care and impacts individuals’ personal and financial lives.^2^^,^^3^ Pain can be classified according to its time of evolution (acute or chronic), its site of origin (peripheral, central, visceral, and somatic), and its pathophysiological mechanism (neuropathic, nociplastic, and nociceptive).^3^^,^^4^ When observed clinically, it is possible to identify that pain triggers a wide variety of motor adaptations, ranging from subtle motor compensations during the performance of tasks to the complete avoidance of painful movements and activities.^2^

Chronic pain (CP) is characterized by its persistence after three months of the typical recovery period from an injury, or by being associated with chronic pathological conditions, leading to continuous or recurrent pain.^4^^,^^5^ Furthermore, CP is classified as a disease by the International Classification of Diseases - 11, entitled primary chronic pain (that is not explained by another chronic condition), there are also secondary chronic pains, which are related to other pathologies or conditions (CP related to cancer, neuropathic CP, secondary visceral CP, secondary musculoskeletal CP, secondary post-surgical/post-traumatic CP or secondary headache/orofacial CP).^5^ CP is considered an important public health problem, with serious consequences for both the individual and society in personal, social and economic terms, and may also be associated with higher levels of physical and emotional stress. Furthermore, it has a higher prevalence in women between the ages of 45 and 65.^4^^,^^5^ CP interferes with the ability to work, since it is one of the main causes of disability. A study conducted with North American citizens estimated that the costs of people with chronic pain were around US$560 billion per year in medical costs and lost productivity.^3^ CP is the main cause of sick leave, absenteeism and low productivity in the workplace.^4^

Currently, there is a growing increase in the prevalence of work-related musculoskeletal disorders (WMSDs) in several countries, manifesting in different clinical forms and reaching epidemic proportions. In the United States, WMSDs are the main cause of pain, suffering and disability in the workplace.^6^ WMSDs affect workers in various occupations, and this set of disorders affects muscles, tendons and nerves. The most common risk factors are poor posture and forced repetitive tasks; The presence of these disorders usually presents with insidious pain that, if left untreated, can lead to temporary or permanent incapacity for work.^7^ Work-related pain is directly linked to the increased number of sick leaves and absenteeism, and is the leading cause of disability, socioeconomic problems and reduced quality of life in the adult population of developed countries. Workers in various occupations have their health affected by debilitating musculoskeletal pain and/or work-related injuries in the hospital environment; musculoskeletal diseases continue to be the leading cause of decline in the workforce.^7^^,^^8^

Nursing professionals, in particular, are at greater risk than other health professionals of experiencing work-related musculoskeletal injuries and disorders, including low back pain.^7^^,^^8^ In nursing professionals, ergonomic factors, such as patient handling and other activities related to manual patient repositioning, have been identified as major risk factors for the presence of pain and injuries for these professionals, especially in the lumbar spine region.^9^ Nursing staff constitute the largest group of workers in the hospital setting and are responsible for the majority of patients’ care.^10^ During their duties, nursing professionals have a high physical burden; the continuous and repetitive action of lifting and transferring patients, associated with physical limitations due to poor ergonomics of hospital equipment, results in greater physiological stress for these professionals.^8^^,^^9^

The presence of disabling pain in this population requires attention, since nursing professionals are indispensable for the provision of quality health care and are present at all levels of health care. According to the Federal Nursing Council (COFEN), it is estimated that this category is responsible for approximately 90% of the care processes carried out in the health area, as well as for 60 to 80% of all actions in Primary Health Care.^11^ Disabling pain can lead to an increase in sick leave and absenteeism among these professionals, causing a workforce deficit to provide health care to patients. A preliminary search was carried out in the following databases: PROSPERO, PubMed, Cochrane Database of Systematic Reviews and Open Science Framework and no published or ongoing scoping and systematic reviews were found that address the prevalence of pain manifestations in nursing professionals. Based on this assumption, it was observed that there is a need for an overview of the prevalence of pain in nursing professionals. That said, this review aims to map the literature on the prevalence of pain in nursing professionals. 

## Methods

This scoping review was conducted according to the Joanna Briggs Institute (JBI) methodology for scoping reviews,^12^ and according to the Preferred Reporting Items for Systematic Reviews and Meta-Analyses extension for Scoping Reviews (PRISMA-ScR)^13^ for preparing the review report. The protocol for this review was developed and registered in the Open Science Framework (OSF) [https://osf.io/2zu73/]. The guiding question for this review was developed based on the mnemonic PCC, Population (nursing professionals), Concept (pain) and Context (work environment). Thus, this scoping review aims to answer the research question: What is the prevalence of pain in nursing professionals?

### Eligibility criteria

The inclusion criteria were established based on the elements of the guiding question, according to the PCC mnemonic, as detailed below: (P) - Population: studies that assessed the prevalence of pain in nursing professionals, whether nursing technicians, nursing assistants or nurses; (C) - Concept: manifestations of pain presented by nursing professionals; and (C) - Context: work environment. In view of the above, studies were included regardless of the year of publication and in all languages, with a view to developing a complete review with relevant quality. The following were excluded: studies that did not fit the research theme, studies that assessed other health professionals, qualitative studies, review studies, reports, protocols, letters, comments and conference proceedings.

### Search strategy

The search strategy aimed to locate published and unpublished studies. As recommended by the JBI (REF), the search process was carried out in 3 phases in the development of a comprehensive research strategy.

First phase: conducting an initial limited search in a selected database, with the aim of finding articles related to the topic of interest. In this first stage, the PubMed database was chosen. In this initial search, the following descriptors and Boolean operators were used: pain AND nurses AND occupational diseases. Based on the initial result (n=700), the titles, abstracts and index terms used to describe and categorize them were read. When observing that the initial strategy presented high sensitivity and many of the studies did not meet the inclusion criteria, the necessary adjustments were made to perform a new search in the same database, using the descriptors: Nurse, Occupational Disease, Musculoskeletal Disease, Ache, Physical Suffering. The result of the new search was 272 articles. The titles and abstracts of the first 20 articles were read to determine whether they would be relevant to the guiding question of the review. After observing that the new strategy proved to be more appropriate, adaptations were made for the other databases. The final search strategies are detailed in Table 1, with the respective adaptations for each of them.

**Table 1:** Search strategies

Database	Search strategy	Result
PubMed	((((nurse) AND (occupational disease)) AND (Musculoskeletal Disease)) AND (ache)) AND (Physical Suffering)	272
BVS	(nurses) AND (pain) AND (occupational disease) AND (Physical Suffering)	83
*Web of Science*	((ALL=(nurse)) AND ALL=(pain)) AND ALL=(occupational disease)	264
SciELO	(pain) AND (nurses) AND (occupational diseases)	11
*Scopus*	(TITLE-ABS-KEY ( nurse ) AND TITLE-ABS-KEY ( occupational AND disease ) AND TITLE-ABS-KEY ( musculoskeletal AND disease ) AND TITLE-ABS-KEY ( pain ) )	333
Embase	('nurse'/exp OR nurse) AND 'occupational disease' AND 'musculoskeletal disease' AND pain	64
CAPES Theses and Dissertations Catalogue	(nurse) AND (pain) OR (Physical Suffering) AND (occupational disease)	15

Second phase: it involves conducting targeted researches in each of the selected databases and information sources, as previously defined in the protocol. The following databases were investigated: PubMed/MEDLINE, Virtual Health Library (VHL), Web of Science, Scientific Electronic Library Online (SciELO), SciVerse Scopus, Embase and the Catalog of Theses and Dissertations of the Coordination for the Improvement of Higher Education Personnel (CAPES).

Third phase: scanning the reference lists of the selected studies for critical evaluation, in order to identify any additional relevant research.

### Study selection

After the search, all identified citations were imported into the bibliography management software EndNote Web® and duplicate studies were removed. The remaining articles were imported into Rayyan Systems Inc. (Qatar Computing Research Institute, Doha, Qatar).^15^^,^^16^ After a pilot test, two independent reviewers screened the titles and abstracts according to the eligibility criteria for the review. Potentially relevant studies were retrieved in full and their citation details were imported into Rayyan Systems Inc. Any discrepancies were resolved by a third reviewer. The analysis of gray literature resulting from dissertations or theses occurred without the aid of automated tools. Two independent researchers performed the analysis of the titles and abstracts directly in the CAPES Dissertation and Theses Catalog. When necessary, the authors or coordinators of the graduate programs were contacted to request the full studies. Dissertations or theses that met the eligibility criteria were evaluated in full. The reasons for exclusion of articles, dissertations or theses after reading the full text were described in the PRISMA-ScR flowchart.^13^

### Data extraction

Data were collected using the instrument suggested by the JBI.^12^ Subsequently, these data were standardized and organized in an electronic spreadsheet that included information on the title, authors, year of publication, type of study, number of participants, study location and prevalence of pain presented by nursing professionals.

## Results

**Figure 1:**
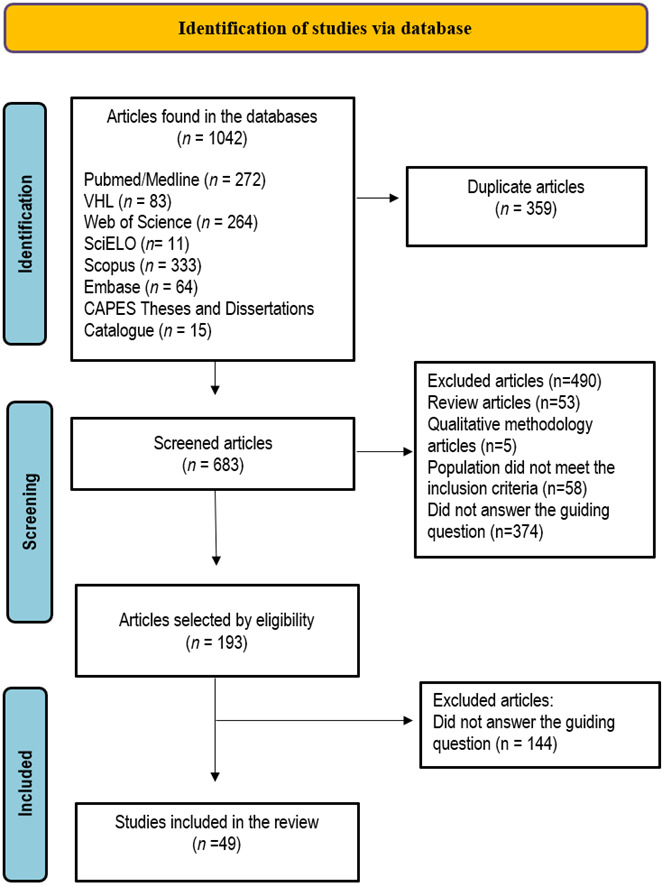
Flowchart of study selection according to PRISMA-SCR.

A total of 1042 studies were identified in the databases, of which 359 were duplicates, totaling 683. After analysis, 490 studies that did not meet the selection criteria were excluded. 193 studies were eligible, and after reading them in full, 144 studies were excluded. Thus, 49 studies were selected for this review. The results of the search and the study inclusion process are described in the PRISMA-SCR flowchart (Figure 1). The studies were published from 1996 to 2023. All were cross-sectional studies and the total sample of nursing professionals included was 35,069. Most of the included studies were concentrated in the Asian continent (71.4%) ^18^^-^^26^^,^^29^^,^^33^^,^^35^^,^^36^^,^^38^^,^^39^^,^^42^^,^^44^^-^^49^^,^^51^^,^^52^^-^^58^^,^^60^^-^^64^ followed by the European (14.2%) ^30^^-^^32^^,^^34^^,^^37^^,^^40^^,^^50^ American (8.1%) ^28^^,^^41^^,^^43^^,^^65^ and African (6.1%) ^17^^,^^27^^,^^59^ continents, with a predominance of publications in the last ten years (Table 2).

**Table 2:** Characteristics of included studies by author, year of publication and results

Author, year of publication, country and sample	Results			
Akodu *et al*., 2019^17^ Nigeria, *n*= 135	Prevalence of WMSD in 12 months:			
	• Low back region: 43.2% • Knees: 9.9% • Shoulders: 9.9%	• Thoracic region: 9.9% • Neck: 8.6% • Elbows: 6.1%	• Ankles: 3.7% • Hips/thighs: 4.9%	• Wrist: 2.5% • Fingers: 1.2%
Almaghrabi *et al*., 2021^18^ Saudi Arabia, *n*=234	82.9% of nurses complained of low back pain.		
	• 0 day: 14.4% • 1 to 7 days: 50%	• 8-30 days: 8.2%	• >30 days: 19.6%	• Daily: 7.7%
Almhdawi *et al*., 2020^19^ Jordan, *n*= 597	Prevalence of WMSD in the lower quadrant in 12 months:		
	• Low back region: 77.4%	• Knee: 37.5%	• Ankle/foot: 28.5%	• Hips/thighs: 22.3%
Amin *et al*., 2014 ^20^ Malaysia *n*= 376	Prevalence of WMSD in 12 months by body region:		
	• Neck: 48.94% • Shoulder: 36.94% • Low back region: 35.28%	• Thoracic region: 40.69% • Arms: 6.63% • Wrists: 26.33%	• Thighs: 19.36% • Knees: 25.55%	• Feet: 47.2% • At least 1 region: 73.2%
Ando *et al*., 2000^21^ Japan, *n*= 314	Prevalence of pain in the last month:		
	• Low back region: 57.7%	• Shoulder: 42.8%	• Neck: 31.3%	• Arm: 18.6%
Attar *et al*., 2014^22^ Saudi Arabia, *n*= 200	The overall 12-month prevalence of self-reported WMSD was 85%.		
	• Low back region: 65.7% • Ankle/foot: 41.5% • Wrist/hand: 10%	• Shoulder: 29% • Knee: 21%	• Neck: 20% • Hips/thighs: 16.5%	•Mid back: 5% •Elbow: 3%
Barzideh *et al*., 2014^23^ Iran, *n*= 385	Regions of musculoskeletal symptoms in the last 12 months:		
	• Low back region: 61.8% • Legs/feet: 59.7%	• Knees: 54.8% • Thoracic region: 54%	• Neck: 48.6% • Wrists/hands: 48.1%	• Shoulders: 45.5% • Thighs: 29.1%
Chandralekha *et al*., 2022^24^ India, *n*= 207	The prevalence of WMSD among those in the last 12 months was 81.2%		
	• Low back region: 55.1% • Neck: 43.5%	• Shoulders: 43%	• More than one region: 38.2%	• More than 6 regions: 18.8%
Cheung *et al*., 2005^25^ China, *n*= 406	The overall prevalence of back pain was 71.2%.		
	• Neck: 62.9% • Shoulders: 73.1% • Back: 71.2%	• Thoracic region: 61.2% • Low back region: 55.9% • Knees: 65.1%	• Ankles/feet: 53.4% • Wrists/hands: 30.3%	• Hips or thighs: 27.7% • Elbows: 17.3%
Cheung *et al*.,2018^26^ China, *n*= 440	Prevalence of WMSD symptoms at the time of the survey:		
	• At least 1 region: 88.4% • Shoulders: 53% • Low back region: 41.4%	• Knees: 37.5 % • Ankles/feet: 28.2 % • Elbows/forearms 27.5%	• Wrists/hands: 25.8% • Fingers 25.7% • Neck: 24.8%	• Calf: 17.7% • Hips/thighs: 11.6% • Thoracic region: 6.4%
Chiwaridzo *et al*., 2018^27^ Zimbabwe, *n*= 117	82.1% reported WMSD in the last 12 months.		
	• Back (lower and upper): 84.3%	• Low back region: 67.9%	
Daraiseh *et al*., 2010^28^ USA, *n*= 263	Musculoskeletal symptoms at 1 month were more prevalent in the following regions:		
	• Low back region: 74.1%	• Neck: 55.2%	• Ankle/foot: 52.5%	• Shoulders: 50%
Dhas *et al*., 2023^29^ , Qatar *n*= 127	Presence of pain reported by region of the body:		
	• Low back region: 55.2% • Neck: 35.5% • Shoulder: 33.9%	• Thoracic region: 29.2% • Wrist/hand: 17.4%	• Ankle/foot: 15.8% • Knee: 15%	• Hips/thighs: 11.9% • Elbow: 7.9%
Engels *et al*., 1996^30^ The Netherlands, *n*= 846	Complaints of pain by body region:		
	• Thoracic region: 7.9% • Low back region: 33.8% • Arm/Neck: 30.4%	• Neck: 22.9% • Shoulder: 19.5% • Elbow: 2.3%	• Wrist/hand: 5.7% • Leg: 15.7% • Hips/thighs: 6.9%	• Knee: 10.2% • Ankle/foot: 3.7%
Eriksen *et al*., 2003^31^ Norway, *n*= 6.485	Prevalence of musculoskeletal pain during the last 14 days:		
	• Head: 41.9% • Neck: 53.5% • Shoulder: 47.1%	• Elbow: 11.7% • Wrist/hand: 20.8% • Thoracic region: 27.3%	• Low back region: 54.9% • Hips/thighs: 26.6% • Knee: 20.5%	• Ankle/foot: 15.5% • Any region: 88.8% • Generalized pain: 26.6%
Freimann *et al*., 2016^32^ Estonia, *n*= 409	Prevalence of musculoskeletal pain in the last year and in the last month:		
	• Low back region: 56.9% • Neck: 55.7%	• Shoulder: 30.9% • Elbow: 12.4%	• Wrist/hand: 20% • Knee: 31.2%	• Any region: 70%
Gaowgzeh, 2019^33^ Saudi Arabia, *n*= 60	61.7% of nurses had low back pain.		
	• Strong: 9.5%	• Moderate: 42.9%	• Mild: 47.6%
Gilchrist *et al*., 2021^34^ Czech Republic, *n*=569	• 84.7% of participants reported low back pain during the previous 12-month period • 76.6% of participants reported low back pain during the previous month		
Karki *et al*., 2023^35^ Nepal, *n*= 165	Prevalence of MSD in the last 12 months:		
	• Neck: 60% • Shoulders: 45.5% • Elbows: 7.3%	• Wrists/hands: 43% • Thoracic region: 51.5%	• Low back region: 75.8% • Hips/thighs: 35.2%	• Knees: 38.8% • Ankles/feet: 37%
Khan *et al*, 2019^36^ Pakistan, *n*= 254	- 185 nurses presented low back pain • 33.46% for more than 10 years • 23.23% for 6 to 10 years • 15.35% for 1 to 5 years. • 0.79% for less than 1 year.	- Among those who worked 6 to 7 hours: • 5.11% had mild pain • 12.6% had moderate pain • 10.63% had moderate pain	- Among those who worked 7 to 8 hours: • 9.06% had mild pain • 23.23% had moderate pain • 12.2% had moderate pain
Knibbe *et al*., 1996^37^ The Netherlands, *n*= 355	Prevalence of back pain:		
	• Last 12 months: 66.8%	• Last 3 months: 51.8%	• Last 7 days: 20.6%
Koğa *et al*., 2019^38^ Türkiye, *n*= 253	• 62.8% of nurses reported a family history of low back pain. • Lifetime prevalence of severe low back pain: 28.2% • Lifetime prevalence of ongoing low back pain: 21.1%		
Krishnan *et al*., 2021^39^ Malaysia, *n*= 300	Complaints of musculoskeletal pain or discomfort reported by nurses over a 12-month period:		
	• Low back: 86.7%; • Ankle/feet: 86.7%; • Neck: 86%;	• Shoulders: 85.3%; • MMI: 85%; • Cervical spine: 84.3%;	• Knees: 77.3%; • Femoral region: 73.7%; • Hip (66.3%);	• Wrist/hand: 63%; • Forearm: 61.7%; • Elbow: 55%;
Latina *et al*., 2020^40^ Italy, *n*= 280	Reports of pain in the last 12 months:		
	• Low back region: 83.4% • Neck: 71.3% • Shoulders: 64.5%	• Back: 59.6% • Wrist: 43.9%	• Knees: 41.9% • Hips/thighs: 39.6%	• Ankles: 29.4% • Elbows: 24.2%
Machado *et al*., 2014^41^ Brazil, *n*= 309	Low back pain was the most frequent health problem reported by professionals (52.8%).		
Mehrdad *et al*., 2010^42^ Iran, *n*= 317	Musculoskeletal symptoms in the last 12 months:		
	• Low back region: 73.2% • Neck: 46.3% • Shoulders: 48.6%	• Elbows: 16.6% • Thoracic region: 43.5%	• Wrists/hands: 42.2% • Ankles/feet: 39.3%	• Hips/Thighs: 28.8% • Knees: 68.7%
Moreira *et al*., 2014^43^ Brazil, *n*= 258	Musculoskeletal symptoms in the last 12 months:		
	• At least 1 region: 93.5% • Cervical spine: 47.8% • Thoracic spine: 50.8% • Lower back spine: 57.1%	• Spine: 76.3% • Shoulder: 52% • Elbow: 7.8%	• Hip/thigh: 32.7% • Wrist/hand: 31.8% • Upper limb: 62%	• Knee: 31.8% • Ankle/foot: 40.4% • Lower limb: 65.3%
Nasaif *et al*., 2023^44^ Bahrain, *n*= 550	The prevalence of musculoskeletal complaints in the last 12 months was 88.1%.		
	• Low back region:72.3%	• Shoulders: 52.8%	• Neck: 49.0%	• Elbow: 12.1%
Nguyen *et al*., 2020^45^ Vietnam, *n*= 1.179	Prevalence of musculoskeletal symptoms during the last 12 months:		
	• Neck: M: 36.2% / W: 45.2% • Shoulder/arm: M: 22.2% / W: 30.6% • Elbow/forearm: M: 5.9% / W:30.6%	• Wrist/hand: M: 8.1% / W: 18.4% • Cervical spine: M: 24% / W: 33.4% • Lower back spine: M:28.5% / W: 47.9%	• Hip/thigh: M: 3.2% / W: 6.5% • Knee/leg: M: 14% / W: 21.4% • Ankle/foot: M: 16% / W: 8.8%
Nourollahi *et al*., 2018^46^ Iran, *n*= 80	Prevalence of WMSD in the body regions of hospital nurses:		
	• Low back region: 72% • Knees: 62% • Cervical spine: 57%	• Legs: 61% • Hands/wrist: 55%	• Neck: 46% • Shoulders: 42%	• Elbows: 30% • Hips: 21%
Pinnar, 2010^47^ Türkiye, *n*= 2.400	Prevalence of WRMD in 12 months by body regions		
	• Low back region: 49.7% • Cervical region: 19.2%	• Neck: 35% • Shoulders: 38%	•Back/neck/shoulders: 13.7% •Legs: 30%	• Any region: 79.5%
Samaei *et al*., 2017^48^ Iran, *n*=243	The prevalence of low back pain among 243 nursing professionals in Iran in the last 12 months was 69.5%.		
Senthilkumar *et al*., 2019^49^ India, *n*= 100	Prevalence of pain in body parts:		
	*ICU Nurses:* • Neck: 57.6% • Shoulders: 44% • Back: 40.2% • Legs: 30.1%	*General ward nurses*: • Neck: 42.1% • Shoulders: 35.6% • Back: 30.6% • Legs: 25.4%	
Serranheira *et al*., 2012^50^ Portugal, *n*= 2.140	- Prevalence of pain symptoms in the last 12 months: • Low back region: 60.6% • Neck: 48.6% • Thoracic region: 44.5%	- Prevalence of pain symptoms in the last 7 days: • Low back region: 29.5% • Neck: 25.8% • Thoracic region: 21.1%	
Sezgin *et al*., 2015^51^ Türkiye, *n*= 1.515	The prevalence of MSD by body regions:		
	• Legs: 64.4% • Low back region: 58.8% • Back: 44.6%	• Shoulders: 33.7% • Neck: 30.3%	• Feet: 14.9% • Arms: 14.6%	• Fist: 9.6% • Head: 7.4%
Sharma *et al*., 2022^52^ India, *n*= 260	The prevalence of WMSD in the last 12 months among Indian nurses was 80%		
	• Neck: 36% • Shoulders: 32%	• Elbow: 5% • Wrists/hands: 10%	• Back: 52% • Hip: 25%	• Knee: 28% • Ankles/Feet: 46%
	Frequency of pain:	• Regular: 50%	• Occasionally: 25%	• Never: 25/%
Shieh *et al*., 2016^53^ China, *n*= 788	72% of study participants reported having low back pain.		
Smith *et al*., 2003^54^ Japan, *n*= 305	Prevalence of MSD:		
	• Low back region: 59% • Neck: 27.9% • Shoulders: 46.6% • Thoracic region: 10.2%	• Arms: 2.6% • Elbows: 2% • Forearms: 1.6%	• Wrists: 4.3% • Thighs: 11.8% • Knees: 16.4%	• Legs: 8.5% • Ankles: 7.5% • Any region: 78.4%
Smith *et al*., 2004^55^ China, *n*= 282	Prevalence of musculoskeletal complaints in the last 12 months:		
	• Any region: 70% • Low back region: 56%	• Neck: 45%	• Shoulder: 40%	• Thoracic region: 37%
Smith *et al*., 2004^56^ China, *n*= 206	MSD prevalence in the 12-month period:		
	• Low back region: 56.7% • Neck: 42.8% • Thoracic spine: 38.9%	• Shoulder: 38.9% • Elbows: 10% • Knees: 31.1%	• Wrists: 27.8% • Legs: 22.8%	• Ankle/feet: 34.4% • Any region: 70%
Smith *et al*., 2005^57^ Korea, *n*= 330	Prevalence of musculoskeletal symptoms:		
	• Neck: 62.7% • Shoulders: 74.5% • Thoracic region: 29.7%	• Elbow: 6.4% • Forearm: 9.7% • Wrists/hands: 46.7%	• Low back region: 72.4% • Thighs: 14.2% • Knees: 35.2%	• Legs: 52.1% • Feet: 38.8% • Any region: 93.6%
Tang *et al*.,2022^58^ China, *n*= 651	Twelve-month prevalence of SCI:		
	• Low back region: 73.5% • Neck: 73.2% • Shoulders: 66.2%	• Thoracic region: 56.3% • Thighs/hips: 38.9%	• Elbows: 29.5% • Wrists/hands: 42.6%	• Knees: 42.3% • Ankles/feet: 42.5%
Tinubu *et al*., 2010^59^ Nigeria, *n*= 128	Prevalence of musculoskeletal disorders:		
	• Low back region: 44.1% • Neck: 28% • Knees: 22.4%	• Thoracic region: 16.8% • Wrists/hands: 16.2%	• Shoulders: 12.6% • Ankles/feet: 10.2%	• Elbows: 7.1% • Hips/thighs: 3.4%
Tojo *et al*.,2018^60^ Japan, *n*= 640	The prevalence of foot and ankle pain in the last month was 23% (SNQ) and 51% (MFPDI).		
	• Hallux: 14% • Little toe: 14% • Plantar forefoot: 9%	• Medial arch: 9% • Midfoot: 16%	• Ankle: 10% • Heel: 6%	• Heel back: 7% • Overall: 23%
Yan *et al*., 2017^61^ China, *n*= 6674	- Prevalence of WMSD in the last 12 months:		
	• Neck: 59.77% • Shoulder: 49.66% • Back: 39.5%	• Elbow: 14.49% • Low back: 62.71% • Wrists: 21.7%	• Hip: 20.41% • Knee: 33.35% • Ankle: 29.86%	• 1 body region: 77.43% • 2 body regions: 68%
Yang *et al*., 2019^62^ China, *n*= 679	Prevalence of pain by body region in the last 12 months:		
	• Low back region: 80.1% • Neck: 78.6% • Shoulder: 70.4%	• Thoracic spine: 39.3% • Elbow: 15.8% • Wrist/hand: 38.9%	• Hip/thigh: 29.9% • Knee: 37.4%	• Ankle/foot: 31.5% • Overall WMSD: 97.1%
Yao *et al*., 2019^63^ China, *n*= 692	Prevalence of WMSD in the last 12 months:		
	• Elbow: 17.3% • Hip: 23.8% • Knee: 34.5%	• Hands/wrists: 30.1% • Ankle/foot: 30.6% • Neck: 68.2%	• Back: 39.7% • Shoulder: 54.6%	• Waist: 67.6% • Any region: 84%
Yilmaz *et al*., 2022^64^ Türkiye, *n*= 169	Pain regions:		
	• Low back region: 68% • Neck: 52.1% • Back: 68%	• Shoulder: 46% • Elbow: 10.7%	• Hands/wrists: 29.6% • Hips/thighs: 28.4%	• Knees: 37.3% • Foot/ankle: 41.4%
Zhang *et al*., 2020^65^ USA, *n*= 327	Reports of pain in the following areas of the body:		
• Low back region: 63% • Neck: 50.6%	• Shoulder: 42.4% • Knee: 35%	• Fist/forearm: 24.2%	• Ankle/foot: 39.3%.

Legend: MSD - Musculoskeletal disorders; WMSD - Work-related musculoskeletal disorders; RSI - Repetitive strain injury; SCI - Musculoskeletal injuries; ADL - Activities of daily living; SNQ - Standardized Nordic Questionnaire; MFPDI - Manchester Foot Pain and Disability Index.

## Discussion

This scoping review mapped the literature on the prevalence of pain in nursing professionals. Among the selected studies that evaluated multiple regions of the body (38 studies)^17^^,^^19^^-^^26^^,^^28^^-^^32^^,^^35^^,^^39^^,^^40^^,^^42^^-^^47^^,^^49^^-^^52^^,^^54^^-^^59^^,^^61^^-^^65^, the majority demonstrated that the most affected area was the low back region (81.57%), followed by the neck (71.5%) and shoulder (31.57%) regions. Eight studies evaluated only the prevalence of low back pain^18^^,^^33^^,^^34^^,^^36^^,^^38^^,^^41^^,^^48^^,^^53^, two of back pain ^27^^,^^37^ and one only pain in the foot and ankle region.^60^ The incidence of low back pain among hospital nursing professionals is considerably high, being the main reason for sick leave in this professional segment.^76^ High physical or mechanical demands strain and fatigue the muscles, which can trigger low back pain due to prolonged positions and repetitive movements.^73^ Low back pain is recognized as a significant occupational risk in most countries, causing long-term impacts on the health of nurses, compromising their work performance and job stability, and having an overall impact on the quality of care provided to patients.^74^ In addition, nurses who have had low back pain and continue to work are at greater risk of experiencing situations that aggravate their low back pain.^75^

In all included studies, the pain was of musculoskeletal origin. Musculoskeletal disorders (MSDs) represent a major health concern, being internationally recognized as the second leading cause of physical disability.^66^ MSDs represent a significant problem for nursing professionals, since they directly impact quality of life, increase absenteeism and restriction of work functions, in addition to the considerable financial cost for individuals and organizations.^59^ Some studies have concluded that the high prevalence of pain in various regions of the body was associated with psychosocial factors, especially stress, suggesting that the interactions of psychosocial factors and physical exhaustion potentially increased the risk of musculoskeletal pain in nursing professionals.(^20,42,55)^ In addition, another study indicated that high psychological and physical demands at work were associated with an increase in back injuries.^23^

Musculoskeletal pain symptoms have also been associated with organizational factors, such as type of hospital, frequent schedule changes, type of shift work, patient handling, and working conditions,^21^^,^^49^^,^^51^ similarly, high physical workload is associated with increased low back pain in hospital nurses, along with longer working hours and many hours standing with constant commuting.^18^^,^^22^^,^^25^^,^^26^^,^^53^^,^^59^ High workloads and stress have been associated not only with pain but also with the risk of injuries. In the study by Clark^67^ *et al*., it was observed that high work demands were related to an increased likelihood of needlestick injuries and near misses among hospital nurses.^67^ Musculoskeletal pain in nurses is a result of work demands and physical exhaustion resulting from the professional activities performed, mainly affecting the low back, thoracic, and cervical regions.^68^^-^^70^

In the study by Tojo *et a*l.^60^, where the prevalence of pain in the foot and ankle region was investigated, a pain prevalence rate in the last month of 23% was found using the standardized Nordic questionnaire and 51% using the Manchester Foot Pain and Disability Index. Other studies included also provided data on prevalence in the foot and ankle region, where in four of them, the prevalence rate was greater than 50%^23^^,^^25^^,^^28^^,^^39^. Several conditions can generate chronic pain in the feet and ankle, however, the pain is mainly caused by inadequate footwear.^71^ Another important factor is work activities, since nurses spend most of their working time standing, they can develop several conditions resulting from the use of inadequate footwear, which can cause pain in the feet and ankles, such as plantar fasciitis, bunions and hammertoes. ^(^^72^

Work-related musculoskeletal disorders in nursing staff arise from direct activities with patients, such as bed baths, adjusting patients in bed, changing clothes, and transferring patients between beds and stretchers. This occurs when appropriate techniques are not used to deal with repetitive, monotonous, and physically demanding activities.^77^ Additionally, rotating shifts and night work contribute to pain, fatigue, and illness among these professionals.^78^

The conclusion of this study is that the prevalence of pain in nursing professionals was of musculoskeletal origin, with the most affected areas being the low back, neck, and shoulder regions. Working conditions, long working hours, and the intense workload faced by nursing professionals are associated with the presence of pain, especially in the low back region. The high prevalence of pain found reinforces the importance of monitoring the health of nursing workers, as well as the need for occupational changes, preventive actions and health education, since the presence of pain affects the well-being of the physical and occupational health of the nursing professional, which can compromise the quality of care and increase absenteeism at work.
